# A new multi-attribute decision making approach based on new score function and hybrid weighted score measure in interval-valued Fermatean fuzzy environment

**DOI:** 10.1007/s40747-023-01021-7

**Published:** 2023-03-20

**Authors:** Hongwu Qin, Qiangwei Peng, Xiuqin Ma, Jianming Zhan

**Affiliations:** 1 College of Computer Science and Engineering, Northwest Normal University, Lanzhou, Gansu China; 2 Department of Mathematics, Hubei Minzu University, Enshi, Hubei China

**Keywords:** Interval-valued Fermatean fuzzy sets, Fuzzy sets, MADM, Score function

## Abstract

Interval-valued Fermatean fuzzy sets (IVFFSs) were introduced as a more effective mathematical tool for handling uncertain information in 2021. In this paper, firstly, a novel score function (SCF) is proposed based on IVFFNs that can distinguish between any two IVFFNs. And then, the novel SCF and hybrid weighted score measure were used to construct a new multi-attribute decision-making (MADM) method. Besides, three cases are used to demonstrate that our proposed method can overcome the disadvantages that the existing approaches cannot obtain the preference orderings of alternatives in some circumstances and involves the existence of division by zero error in the decision procedure. Compared with the two existing MADM methods, our proposed approach has the highest recognition index and the lowest error rate of division by zero. Our proposed method provides a better approach to dealing with the MADM problem in the interval-valued Fermatean fuzzy environment.

## Introduction

This is a serious challenge to face up to the uncertain and fuzzy data in real-life applications involving many fields such as industry, environment science, and engineering so on. Atanassov was the first to propose the intuitionistic fuzzy sets (IFSs) [1, 2]. IFSs are very efficient and useful mathematical techniques for dealing with ambiguous data [3–5], which are represented by the membership degree (MSD) and non-membership degree (NMSD), and also have the restriction that$\ 0\leqslant MSD+NMSD \leqslant 1$ [6]. Atanassov and Gargov established the notion of interval-valued intuitionistic fuzzy sets (IVIFSs) in order to overcome the constraints of prior IFS-based approaches to dealing with fuzzy data [7]. From then on, IVIFSs have been employed in a variety of contexts, including decision making [8–10], optimization [11–13], pattern recognition [14, 15] and image segmentation [16]. To handle more ambiguous information, Yager et al. proposed Pythagorean fuzzy sets (PFSs), which are more adaptable than IFSs because$\ 0 \leqslant MSD^2+NMSD^2 \leqslant 1$ [17, 18]. To be able to flexibly enlarge or reduce the limitation range of intuitionistic fuzzy sets, Yager et al. defined q-rung orthopair fuzzy sets, their limitation is$\ 0 \leqslant MSD^n+NMSD^n \leqslant 1$ [19]. Yager et al. discovered that when q gets bigger, the universe of qualifying orthopairs expands, enabling users to process more ambiguous information [20–22]. When q = 3 in the q-rung orthopair fuzzy sets, Senapati and Yager called these new fuzzy sets as Fermatean fuzzy sets (FFSs) [23]. Then Senapati and Yager [24] defined the basic operation of FFSs, and gave the SCF and accuracy function of FFSs. Inspired by FFSs and IVIFSs, Jeevaraj et al. proposed the notion of interval-valued Fermatean fuzzy sets (IVFFSs) and defined some basic mathematical operations on IVFFSs [25]. The limitation of IVIFSs is$\ 0 \leqslant MSD+NMSD \leqslant 1$, while IVFFSs can deal with the uncertainty that $\ 0 \leqslant MSD^3+NMSD^3 \leqslant 1$. Obviously, IVFFSs can handle a wider range of uncertain information than IVIFSs [26]. Therefore, IVFFS are gradually becoming a more effective mathematical model for dealing with uncertainty problems.

In recent years, numerous MADM approaches proposed in various environment, such as intuitionistic fuzzy environment [27–30]. In Pythagorean fuzzy environment [31, 32], Fermatean fuzzy environment [33–35], interval-valued intuitionistic fuzzy environment [36, 37], interval-valued Fermatean fuzzy environment [38] and other fuzzy environments [39–43]. In intuitionistic fuzzy environment, Senapati et al. [44] proposed some computations and some new aggregation operators, and they used these aggregation operators to address the MADM problem of the worldwide supplier selection and the health-care waste disposal method selection. In [45], Sharma et al. introduced a multi-objective bi-level chance-constrained optimization to solve a company’s production planning problem in intuitionistic fuzzy environment. In Pythagorean fuzzy environment, Soltani et al. [46] developed a Pythagorean fuzzy MADM model to enhance the application process of traditional lean manufacturing. Besides, in [47], Fei and Feng introduced a dynamic multi-attribute decision framework based on PFSs that fuses decision information from different periods for addressing the effect of the time factor on MADM results. In Fermatean fuzzy environment, Saha et al. [48] extended the Delphi techniques to identify attributes based on FFSs and proposed Double Normalized MARCOS approach and constructed a novel MADM model to address the warehouse site selection. Moreover, Hezam et al. [49] presented a hybrid double normalization-based multi-aggregation approach to improve the transportation services for the person with disabilities. In the interval-valued intuitionistic fuzzy environment, Chen and Yu [50] proposed a new MADM approach based on a novel score function and the power operator. In [51], Chen et al. combined the best-worst method, decision-making trial, and evaluation laboratory to construct a MADM approach to solve resumption risk assessment amid COVID-19 in interval-valued intuitionistic fuzzy environment. Rani et al. [52] introduced Einstein aggregation operators and constructed an integrated attribute interaction via inter-criteria correlation and the complex proportional assessment approach under IVFFNs to tackle the MADM problem in interval-valued Fermatean fuzzy environment. Jeevaraj [25] created the TOPSIS approach for tackling MADM problems under the interval-valued Fermatean fuzzy environment. Rani et al. [53] devised the weighted aggregated sum product assessment approach for IVFFNs to handle the MADM problems.

However, the existing score functions in [25, 52–54] can not distinguish two IVFFNs in some situations. And the MADM approaches mentioned in [52, 54] have the shortcomings that the MADM approach proposed in [54] and the MADM approach proposed in [52] cannot derive the preference orderings of alternatives in some situations. To solve the above problems, a novel score function and MADM approach are proposed in this paper.

In detail, our main contributions are as follows:

1. A novel score function is defined to address the shortcoming of existing methods that cannot distinguish between two IVFFNs in some situations.

In contrast to the SCF proposed by Rani and Mishra [53], Jeevaraj [25], Rani et al. [52] and Chen and Tsai [54], our proposed SCF can distinguish between any two IVFFNs. Experimental results demonstrate that our SCF has the highest differentiation rate among these above SCFs.

2. A new MADM approach has been developed based on our proposed SCF in this paper. In contrast to Rani et al.’s MADM approach [52], and Chen and Tsai’s MADM approach [54], our proposed approach can obtain the preference orderings of alternatives in any situation. Three real-life cases show that our MADM method has the highest recognition index among the three MADM methods.

3. When the interval values of the non-membership degree of the IVFFNs in the decision matrix are equal to the interval values of the membership degree, our proposed MADM approach can avoid the error as the existence of division by zero while Rani et al.’s MADM approach [52] involves the existence of division by zero error in the decision procedure. Therefore, our method has the lowest error rate of division by zero as 0, which is illustrated by experiments.

This paper is arranged as follows. “Preliminaries” discusses the fundamental definitions. “Analyzing the deficiencies of the existing MADM approaches” mainly discusses the drawbacks of existing MADM approaches. In “A new MADM approach based on the proposed score function of IVFFNs and the hybrid weighted score measure of IVFFNs”, a new SCF is proposed in interval-valued Fermatean fuzzy environment. Besides, the proposed novel SCF and hybrid weighted score model are used to build a new MADM approach. The new MADM approach can overcome the deficiencies of the MADM approaches described in [52, 54]. “Applications of the proposed MADM method” applies three real-life cases to demonstrate the rationality and superiority of our proposed MADM method. “Comparison with the two existing MADM approaches” demonstrates the superiority of our proposed score function by differentiation rate, and we introduce recognition index and error rate to prove the higher performance of our proposed MADM method compared with existing methods. Finally, “Conclusion” draws concludes with a summary of our work and recommendations for extending our suggested MADM approach to other fuzzy environments.

## Preliminaries

IVFFSs are generalization of FFSs and PFSs. The following are some basic concepts related to IVFFSs.

### Definition 1

[55] Let$\ T={t_1,t_2,...,t_P}$ is a finite domain of discourse. A FFS D in T is defined as follows:

$$\begin{aligned} D=\left\{ \left\langle t,\mu _A\left( t \right) ,v_D\left( t \right) \right\rangle :t\in T \right\} \end{aligned}$$

where$\ \mu _D(t)\in [0,1]$, $\ v_D(t) \in [0,1]$, $\ \left\{ t\in X \mid T \subseteq [0,1]\right\}$, in addition, the cube sum of MSD$\ \mu _D(t)$ and NMSD$\ v_A(t)$ is equal to 1, such that$\ 0<(\mu _A(t))^3+(v_A(t))^3\le 1$. For any FFS A and$\ t\in T$, $\ \pi _D(t)=\root 3 \of {1-(\mu _D(t))^3-(v_D(t))^3}$ is referred to as the degree of reluctance or indeterminacy of t to A.

### Definition 2

[25] Let$\ T={t_1,t_2,...,t_P}$ is a finite domain of discourse, an IVFFS F in T is defined as follows:

$$\begin{aligned} F=\left\{ <t,\mu _F(t),v_F(t)>:t\in T \right\} \end{aligned}$$

where$\ \mu _F(t)\in [0,1]$, $\ v_F(t) \in [0,1]$, $\ \left\{ t\in T \vert T\subseteq [0,1]\right\}$, with the circumstance $\ \forall t\in T$, $\ 0 \leqslant sup\_t(\mu _F(t))^3+sup\_t(v_F(t))^3 \leqslant 1$. The intervals of$\ \mu _F(t)$ and$\ v_F(t)$ represent the degree of t to F’s membership and non-membership, respectively. $\ \forall t \in T$, $\ \mu _F(t)$ and$\ v_F(t)$ are closed intervals and their lower and upper bounds are represented by$\ \mu _F^L(t)$, $\ \mu _F^U(t)$ and$\ v_F^L(t)$, $\ v_F^U(t)$, respectively. Hence, another equivalent expression of an IVFFS F is expressed as

$$\begin{aligned} F=\left\{ <t,[\mu _F^L(t),\mu _F^U(t)],[v_F^L(t),v_F^U(t)]>:t\in T \right\} \end{aligned}$$

where$\ 0<(\mu _F^U(t))^3+(v_F^U(t))^3\le 1$. For each element$\ t\in T$, the degree of uncertainty is defined as

$$\begin{aligned} \pi _F(t )&=[\pi _F^L(t ),\pi _F^U(t )] \\&=\bigg [\root 3 \of {1-(\mu _F^U(t ))^3-(v_F^U(t ))^3},\\&\quad \root 3 \of {1-(\mu _F^L(t ))^3-(v_F^L(t ))^3}\bigg ]. \end{aligned}$$

### Definition 3

[53] Let$\ \tau =([\mu _t^L,\mu _t^U],[v_t^L,v_t^U])$ be an IVFFN, then Rani and Mishra’s SCF M of the IVFFN is depicted as follows:

1 $$\begin{aligned} M(\tau )=\frac{{\mu _t ^L}^3+{\mu _t ^U}^3-{v_t ^L}^3-{v_t ^U}^3}{2} \end{aligned}$$

where$\ M(\tau )\in [-1,1]$. The greater the magnitude of$\ M(\tau )$, then the greater the magnitude of the IVFFN$\ \tau$.

### Definition 4

[53] Let$\ \tau =([\mu _t^L,\mu _t^U],[v_t^L,v_t^U])$ be an IVFFN, then Rani and Mishra’s accuracy function H of the IVFFN is defined as follows:

2 $$\begin{aligned} H(\tau )=\frac{{\mu _t^L}^3+{\mu _t^U}^3+{v_t^L}^3+{v_t^U}^3}{2} \end{aligned}$$

where$\ H(\tau ) \in [0,1]$. The greater the magnitude of$\ H(\tau )$, then the greater the magnitude of the IVFFN$\ \tau$.

### Definition 5

[25] Let$\ \tau =([\mu _t^L,\mu _t^U],[v_t^L,v_t^U])$ be an IVFFN, then Jeevaraj’s precise SCF P of the IVFFN is defined as follows:

3 $$\begin{aligned} P(\tau )=\frac{-{\mu _t^L}^3+{\mu _t^U}^3+{v_t^L}^3-{v_t^U}^3}{2} \end{aligned}$$

where$\ P(\tau )\in [-\frac{1}{2},\frac{1}{2}]$. The larger the score$\ P(\tau )$, the smaller the IVFFN$\ \tau$.

### Definition 6

[25] Let$\ \tau =([\mu _t^L,\mu _t^U],[v_t^L,v_t^U])$ be an IVFFN, then Jeevaraj’s complete SCF C of the IVFFN is defined as follows:

4 $$\begin{aligned} C(\tau )=\frac{-{\mu _t^L}^3+{\mu _t^U}^3-{v_t^L}^3+{v_t^U}^3}{2} \end{aligned}$$

where$\ C(\tau )\in [-\frac{1}{2},\frac{1}{2}]$. The greater the magnitude of$\ C(\tau )$, then the greater the magnitude of the IVFFN$\ \tau$.

### Definition 7

[52] Let$\ \tau =([\mu _t^L,\mu _t^U],[v_t^L,v_t^U])$ be an IVFFN, then Rani et al.’s SCF R of the IVFFN is defined as follows:

5 $$\begin{aligned}&R(\tau )\nonumber \\&\quad =\frac{\begin{matrix}({\mu _t^L}^3-{v_t^L}^3)\bigg (1+\root 3 \of {1-{\mu _t^L}^3-{v_t^L}^3}\bigg )+({\mu _t^U}^3-{v_t^U}^3)\\ \bigg (1+\root 3 \of {1-{\mu _t^U}^3-{v_t^U}^3}\bigg )\end{matrix}}{2} \end{aligned}$$

where$\ R(\tau )\in [-1, 1]$. The greater the magnitude of$\ R(\tau )$, then the greater the magnitude of the IVFFN$\ \tau$.

### Definition 8

[54] Let$\ \tau =([\mu _t^L,\mu _t^U],[v_t^L,v_t^U])$ be an IVIFN, then Chen and Tsai’s SCF G of the IVIFN is defined as follows:

6 $$\begin{aligned} G(\tau )=\frac{\sqrt{\mu _t^L}+\sqrt{\mu _t^U}+\sqrt{1-v_t^L}+\sqrt{1-v_t^U}}{2} \end{aligned}$$

where$\ G(\tau )\in [0,2]$. The greater the magnitude of$\ G(\tau )$, then the greater the magnitude of the IVIFN$\ \tau$.

### Definition 9

[25, 52, 53] Let$\ F=([\mu _F^L,\mu _F^U],[v_F^L,v_F^U])$ and$\ G=([\mu _G^L,\mu _G^U],[v_G^L,v_G^U])$ be two IVFFNs. The sorting approach between the IVFFNs$\ F$ and$\ G$ is defined as follows:

1. If$\ \mu _F^L \geqslant \mu _G^L, \mu _F^U \geqslant \mu _G^U, v_F^L \leqslant v_G^L$ and$\ v_F^U \leqslant v_G^U$ then$\ F\geqslant G$.
2. If$\ \mu _F^L = \mu _G^L, \mu _F^U = \mu _G^U, v_F^L = v_G^L$ and$\ v_F^U = v_G^U$ then$\ F = G$.

## Analyzing the deficiencies of the existing MADM approaches

In this part, we analyze the deficiencies of existing MADM approaches in Chen and Tsai [54] based on IVIFNs and Rani et al. [52] for IVFFNs. IVFFSs is the superset of IVIFSs [25]. That is, the ranking principle on IVFFNs as generalized model still functions for IVIFNs as the subclass. Consider the following multi-attribute decision problem:$\ P= \left\{ p_1,p_2,...,p_m \right\}$ is a group of m plausible alternatives and$\ R=\left\{ r_1,r_2,...,r_n \right\}$ is a finite collection of characteristics. Consider that decision maker gives his own decision matrix $M=( \chi _{ij})_{m\times n}=([\mu _{ij}^L,\mu _{ij}^U],[v_{ij}^L, v_{ij}^U])_{m\times n} (i=1,2,...,m, j=1,2,...,n)$, where $[\mu _{ij}^L,\mu _{ij}^U]\in [0,1], [v_{ij}^L, v_{ij}^U]\in [0,1], 0 \leqslant {\mu _{ij}^U}^3+{v_{ij}^U}^3 \leqslant 1$. Assume the decision maker has provided the following interval-valued Fermatean fuzzy decision matrix $\ M=( \chi _{ij})_{m\times n}=([\mu _{ij}^L,\mu _{ij}^U],[v_{ij}^L,v_{ij}^U])_{m\times n}$:
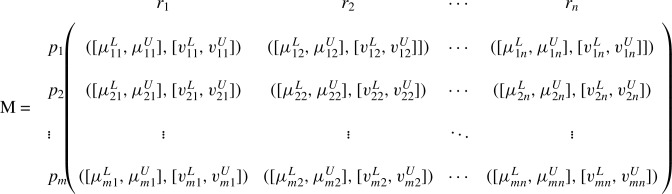


where $\ [\mu _{ij}^L,\mu _{ij}^U]$ is an evaluative IVFFN of the attribute$\ r_j$ relative to the alternative $p_i, 0\leqslant {\mu _t}^L\leqslant {\mu _t}^U \leqslant 1$, $0\leqslant {v_t}^L\leqslant {v_t}^U \leqslant 1$, $0\leqslant {\mu _t^U}^3+{v_t^U}^3 \leqslant 1$, $1\leqslant i \leqslant m$, $1\leqslant j \leqslant n$.

Following is a brief description of Chen and Tsai’s MADM approach [54].

**Step 1:** According to Eq. (6) and the decision matrix$\ M=( \chi _{ij})_{m\times n} =([\mu _{ij}^L,\mu _{ij}^U],[v_{ij}^L,v_{ij}^U])_{m\times n}$, build the score matrix$\ SM =(\sigma _{ij})_{m\times n}$ as follows:

7 $$\begin{aligned} \sigma _{ij}= \left\{ \begin{matrix} G(\chi _{ij}), \quad r_j\in R_1\\ 2-G(\chi _{ij}), \quad r_j \in R_2 \end{matrix}\right. , \end{aligned}$$

where$\ R_1$ is the set of benefit attributes and$\ R_2$ is the set of cost attributes, and$\ G(\chi _{ij})=(\sqrt{\mu _{ij}^L}+\sqrt{\mu _{ij}^U}+\sqrt{1-v_{ij}^L}+\sqrt{1-v_{ij}^U})/2$, ($\ 1 \leqslant i \leqslant m$, $\ 1 \leqslant j \leqslant n$).

**Step 2:** According to the obtained score matrix$\ SM=(\sigma _{ij})_{m\times n}$, build the normalized score matrix$\ {\tilde{SM}}=({\tilde{\sigma }}_{ij})_{m\times n}$,

8 $$\begin{aligned} {\tilde{\sigma }}_{ij}=\frac{\sigma _{ij}}{\sum _{i=1}^{m}\sigma _{ij}}, \end{aligned}$$

where$$\ \leqslant i \leqslant m$$ and$$\ 1 \leqslant j \leqslant n$$.

**Step 3:** Based on Eq. (6) and the IVFFN weight$\ \psi _j=([a,b],[c,d])$ of attribute$\ r_j$, where$\ j=1,2,...,n$, calculate the optimal weight$\ {\tilde{\psi }}$ of attribute$\ r_j$, where$\ {\tilde{\psi }}_j=\frac{\sqrt{a}+\sqrt{b}+\sqrt{1-c}+\sqrt{1-d}}{2}$, $\ {\tilde{\psi }}_j \in [0,2]$ and$\ j=1,2,...,n$.

**Step 4:** According to the normalized score matrix $\ {\tilde{SM}}=({\tilde{\sigma }}_{ij})_{m\times n}$ and the optimal weight$\ {\tilde{\psi }}_j$ of attribute$\ r_j$, build the weighted normalized decision matrix$\ WDM(\eta _{ij})_{m\times n}$,

9 $$\begin{aligned} \eta _{ij}={\tilde{\psi }}_j \times {\tilde{\sigma }}_{ij}, \end{aligned}$$

where$$\ i=1,2,...,m$$ and$$\ j=1,2,...,n$$.

**Step 5:** Based on the obtained weighted normalized decision matrix

$$WDM(\eta _{ij})_{m\times n}$$, calculate the weight score $$ WS(p_i)$$ of alternative $$ p_i$$, shown as follows:

10 $$\begin{aligned} WS(p_i)=\sum _{j=i}^{n}\eta _{ij}, \end{aligned}$$

where$$\ i=1,2,...,m$$.

**Step 6:** Rank the alternatives $$ P= \left\{ p_1,p_2,...,p_m \right\} $$ based on the weighted scores $$ WS(p_1)$$, $$ WS(p_2),...$$, and $$ WS(p_m)$$. The larger the value of $$ WS(p_m)$$, the better the preference ordering of alternative $$ p_i$$, where $$ i=1,2,...,m$$.

### Example 1

Suppose that$\ p_1$ and$\ p_2$ are two alternatives, and$\ r_1$ and$\ r_2$ are two benefit attributes, and$\ \psi _1$ and$\ \psi _2$ are the IVFFNs weight of attributes $r_1$ and $r_2$ provided by the decision maker, respectively, where $\psi _1=$ ([0.20,0.35], [0.10, 0.50]) and $\psi _2=([0.20,0.25],[0.30,0.40])$. Suppose that the decision maker provides the following decision matrix $M=(\chi _{ij})_{2\times 2} =([\mu _{ij}^L,\mu _{ij}^U],[v_{ij}^L,v_{ij}^U])_{2\times 2}$:where$\ 1\leqslant i \leqslant 2$ and$\ 1\leqslant j \leqslant 2$.

**[Step 1]** Based on the SCF given by Chen in Definition 8 and the decision matrix$\ M=( \chi _{ij})_{2\times 2}$ to construct the score matrix$\ SM =(\sigma _{ij})_{2\times 2}$, where$\ r_1$ and$\ r_2$ are two benefit attributes,$\ \sigma _{ij}=G(\chi _{ij})=(\sqrt{\mu _{ij}^L}+\sqrt{\mu _{ij}^U}+\sqrt{1-v_{ij}^L}+\sqrt{1-v_{ij}^U})/2$, $\ \sigma _{11}=1.05$, $\ \sigma _{12}=0.60$, $\ \sigma _{21}=1.05$, $\ \sigma _{22}=0.60$. Therefore, the score matrix can be obtained as follows:**[Step 2]** Based on the score matrix$\ SM =(\sigma _{ij})_{2\times 2}$, build the normalized score matrix$\ {\tilde{SM}}=({\tilde{\sigma }}_{ij})_{2\times 2}$, where$\ {\tilde{\sigma }}_{ij}=\frac{\sigma _{ij}}{\sum _{i=1}^{2}\sigma _{ij}}$, $\ {\tilde{\sigma }}_{11}=\frac{1.05}{1.05+1.05}=0.5$, $\ {\tilde{\sigma }}_{12}=\frac{1.095}{1.095+1.095}=0.5$, $\ {\tilde{\sigma }}_{21}=\frac{1.05}{1.05+1.05}=0.5$, $\ {\tilde{\sigma }}_{22}=\frac{1.095}{1.095+1.095}=0.5$, then**[Step 3]** Based on Eq. (6), convert the two weights$\ \psi _1$ and$\ \psi _2$ from IVFFNs to crisp numbers according to Eq. (6), where$\ \psi _1=([0.20,0.35], [0.10,0.50])$ and$\ \psi _2=([0.20,0.25],[0.30,40])$. Then,$\ \tilde{\psi _1}=1.347$ and$\ \tilde{\psi _2}=1.279$.

**[Step 4]** According to the normalized score matrix $\ {\tilde{SM}}=({\tilde{\sigma }}_{ij})_{2\times 2}$ and crisp weight of attribute$\ \tilde{\psi _1}$ and$\ \tilde{\psi _2}$ to construct the weighted normalized decision matrix$\ WDM=(\eta _{ij})_{2\times 2}$, where$\ \eta _{11}=\tilde{\psi _1} \times {\tilde{\sigma }}_{11}=0.6735$, $\ \eta _{12}=\tilde{\psi _2} \times {\tilde{\sigma }}_{12}=0.6395$, $\ \eta _{21}=\tilde{\psi _1} \times {\tilde{\sigma }}_{21}=0.6735$, $\ \eta _{22}=\tilde{\psi _2} \times {\tilde{\sigma }}_{22}=0.6395$. Then the weighted normalized decision matrix is as follows:**[Step 5]** Based on the weighted normalized decision matrix$\ WDM =(\eta _{ij})_{2\times 2}$ to calculate the weight score $WS(p_i)$, where $\ WS(p_i)=\sum _{j=i}^{2}\eta _{ij}$, $WS(p_1) =0.6735+0.6395=1.313$, $\ WS(p_2)=0.6735+0.6395=1.313$.

**[Step 6]** Based on the value of$\ WS(p_i)$ to rank the preference orderings of alternatives, where$\ WS(p_1)=1.313$ and$\ WS(p_2)=1.313$. Because the weight score $\ WS(p_1)=WS(p_2)=1.313$, the preference orderings of alternatives$\ p_1$ and$\ p_2$ acquired by Chen and Tsai’s MADM approach [54] is “$p_1=p_2$”. However, the decision matrix$\ M=( \chi _{ij})_{2\times 2}$ indicates that the IVFFNs for alternatives$\ p_1$ and$\ p_2$ under each attribute value are different. Therefore, Chen and Tsai’s MADM approach [54] has the deficiencies that this method cannot distinguish the preference orderings of alternatives and cannot help us make decisions well in this situation.

A brief description of Rani et al.’s MADM approach [52] is shown below. Consider the following multi-attribute decision problem:$\ P= \left\{ p_1,p_2,...,p_m \right\}$ is a group of m plausible alternatives and$\ R=\left\{ r_1,r_2,...,r_n \right\}$ is a finite collection of characteristics.

**Step 1:** According to the decision maker’s opinion, construct the decision matrix $M=( \chi _{ij})_{m\times n}=([\mu _{ij}^L,\mu _{ij}^U], [v_{ij}^L,v_{ij}^U])_{m\times n} (i=1,2,...,m, j=1,2,...,n)$, where $[\mu _{ij}^L,\mu _{ij}^U] \in [0,1], [v_{ij}^L,v_{ij}^U]\in [0,1], {\mu _{ij}^U}^3+{v_{ij}^U}^3 \leqslant 1 (i=1,2,...,m), (j=1,2,...,n)$.

**Step 2:** Based on the criteria interaction through inter-criteria correlation (CRITIC) approach to obtain the weight of each attribute. Assume that$\ \varPsi =(\psi _1,\psi _2,...,\psi _n)^T$ be the group of attribute weight with$\ \psi _j \in [0,1]$ and$\ \sum _{j=1}^{n} \psi _j = 1$. The following are the calculation steps to determine the weight of attributes using the CRITIC approach:

**Step 2.1:** According to Eq. (5) and the decision matrix $M=( \chi _{ij})_{m\times n} =([\mu _{ij}^L,\mu _{ij}^U],[v_{ij}^L,v_{ij}^U])_{m\times n}$, build the score matrix$\ SM =(\sigma _{ij})_{m\times n}$, where

11 $$\begin{aligned}{} & {} \sigma _{ij}\nonumber \\{} & {} \quad =\frac{\begin{matrix}({\mu _{ij}^L}^3-{v_{ij}^L}^3)\bigg (1+\root 3 \of {1-{\mu _{ij}^L}^3-{v_{ij}^L}^3}\bigg )+({\mu _{ij}^U}^3-{v_{ij}^U}^3)\\ \bigg (1+\root 3 \of {1-{\mu _{ij}^U}^3-{v_{ij}^U}^3}\bigg )\end{matrix}}{2}\nonumber \\ \end{aligned}$$

where$$\ 1 \leqslant i \leqslant m$$, $$\ 1 \leqslant j \leqslant n$$.

**Step 2.2:** According to the score matrix$\ SM=(\sigma _{ij})_{m\times n}$, build the normalized score matrix$\ {\tilde{SM}}=({\tilde{\sigma }}_{ij})_{m\times n}$, where

12 $$\begin{aligned} {\tilde{\sigma }}_{ij}= \left\{ \begin{array}{ll} \frac{\sigma _{ij}-{\sigma _j^-}}{{\sigma _j^+}-{\sigma _j^-}}, \quad r_j\in R_1\\ \frac{{\sigma _j^+}-\sigma _{ij}}{{\sigma _j^+}-{\sigma _j^-}}, \quad r_j \in R_2 \end{array}\right. , \end{aligned}$$

and$\ R_1$ is the set of benefit attributes and$\ R_2$ is the set of cost attributes, and $\ {\sigma _j^-}=\mathop {\min }\nolimits _i \sigma _{ij}$, $\ {\sigma _j^+}=\mathop {\max }\nolimits _i \sigma _{ij}$, $\ 1 \leqslant i \leqslant m$ and$\ 1 \leqslant j \leqslant n$.

**Step 2.3:** Calculate the standard deviations$\ \xi _j$ of the attribute$\ r_j$, where

13 $$\begin{aligned} \xi _j = \sqrt{\frac{\sum _{i=1}^{m}({\tilde{\sigma }}_{ij}-{\bar{\sigma }}_j)^2}{m}} \end{aligned}$$

and$$\ {\bar{\sigma }}_j=\sum _{i=1}^{m}{\tilde{\sigma }}_{ij}/n$$, $$\ (i=1,2,...,m$$, $$\ j=1,2,...,n)$$.

**Step 2.4:** Determine the relationship$\ \epsilon _{jk}$ between each attribute as follows:

14 $$\begin{aligned} \epsilon _{jk}=\frac{\sum _{i=1}^{m}({\tilde{\sigma }}_{ij}-{\bar{\sigma }}_j)({\tilde{\sigma }}_{ik}-{\bar{\sigma }}_k)^2}{\sqrt{\sum _{i=1}^{m}({\tilde{\sigma }}_{ij}-{\bar{\sigma }}_j)} \sum _{i=1}^{m}({\tilde{\sigma }}_{ik}-{\bar{\sigma }}_k)^2} \end{aligned}$$

where$$\ i=1,2,...,m$$, $$\ j=1,2,...,n$$ and$$\ k=1,2,...,n$$.

**Step 2.5:** Calculate the information quantity$\ \phi _j$ of each attribute as follows:

15 $$\begin{aligned} \phi _j = \xi _j\sum _{k=1}^{n}(1-\epsilon _{jk}). \end{aligned}$$

where$$\ i=1,2,...,m$$, $$\ j=1,2,...,n$$ and$$\ k=1,2,...,n$$.

**Step 2.6:** According to the information quantity$\ \phi _j$ of each attribute, the weight of each attribute is as follows:

16 $$\begin{aligned} \psi _j = \frac{\psi _j}{\sum _{j=1}^{n}\psi _j}. \end{aligned}$$

where$$\ i=1,2,...,m$$ and$$\ j=1,2,...,n$$.

**Step 3:** According to decision matrix$\ M=(\chi _{ij})_{m\times n}$ and interval-valued Fermatean fuzzy Einstein weighted aggregation (IVFFEWA) operator, build the normalized decision matrix ${\tilde{M}}=\tilde{\chi _i}+ \varsigma _i$. if$\ r_{i{l+1}},r_{i{l+2}},...,r_{in}$ is benefit attributes, then$\ \tilde{\chi _i}=IVFFEWA( \chi _{i1}, \chi _{i2},...,\chi _{il})$, $\ \varsigma _i=IVFFEWA( \chi _{i{l+1}}, \chi _{i{l+2}},...,\chi _{in})$, simlarily, if $r_{i{l+1}},r_{i{l+2}}, ...,r_{in}$ is cost attributes, then$\ \varsigma _i= IVFFEWA( \chi _{i{l+1}}, \chi _{i{l+2}},...,\chi _{in})$. And IVFFEWA operator is shown as follows:

17 $$\begin{aligned} \begin{aligned}&IVFFEWA(\chi _{i1},\chi _{i2},...,\chi _{in})\\&=\left( \left[ \root 3 \of {\frac{\prod _{j=1}^{n}(1+{\mu _{ij}^L}^3)^{ \psi _i}-\prod _{j=1}^{n}(1-{\mu _{ij}^L}^3)^{ \psi _j}}{\prod _{j=1}^{n}(1+{\mu _{ij}^L}^3)^{ \psi _j}+\prod _{j=1}^{n}(1-{\mu _{ij}^L}^3)^{ \psi _j}}},\right. \right. \\&\quad \left. \left. \root 3 \of {\frac{\prod _{j=1}^{n}(1+{\mu _{ij}^U}^3)^{ \psi _j}-\prod _{j=1}^{n}(1-{\mu _{ij}^U}^3)^{ \psi _j}}{\prod _{j=1}^{n}(1+{\mu _{ij}^U}^3)^{ \psi _j}+\prod _{j=1}^{n}(1-{\mu _{ij}^U}^3)^{ \psi _j}}} \right] ,\right. \\&\quad \left. \left[ \frac{\root 3 \of {2\prod _{j=1}^{n}{v_{ij}^L}^{ \psi _j}}}{\root 3 \of {\prod _{j=1}^{n}(2-{v_{ij}^L}^3)^{ \psi _j}+\prod _{j=1}^{n}({v_{ij}^L}^3)^{ \psi _j}}},\right. \right. \\&\quad \left. \left. \frac{\root 3 \of {2\prod _{j=1}^{n}{v_{ij}^U}^{ \psi _j}}}{\root 3 \of {\prod _{j=1}^{n}(2-{v_{ij}^U}^3)^{ \psi _j}+\prod _{j=1}^{n}({v_{ij}^U}^3)^{ \psi _j}}}\right] \right) , \end{aligned} \end{aligned}$$

where$\ \psi _j$ is the weight of attribute$\ r_j$ and$\ {\sum _{j=1}^{n}\psi _j}=1(i=1,2,...m, j=1,2,...,n)$.

**Step 4:** Calculate the “relative degree (RD)” of each alternative.

18 $$\begin{aligned} \gamma _i = \theta V(\tilde{\chi _i})+(1-\theta )\frac{\mathop {\min }\nolimits _i V(\varsigma _i)\sum _{i=1}^{t}V(\varsigma _i)}{V(\varsigma _i)\sum _{i=1}^{t}\frac{ \mathop {\min }\nolimits _i V(\varsigma _i)}{V(\varsigma _i)}}, \end{aligned}$$

where$\ t$ is the number of benefit attribute. And the parameter $\theta \in [0,1]$ represents the decision maker’s strategic coefficient. The strategic parameter evaluation is as follows: if$\ \theta <0.5$, then the decision-maker displays negative behavior, and if$\ \theta >0.5$, then the decision-maker displays optimistic behavior, which indicates that the higher degree is related to the weight of benefit-type attribute, and if$\ \theta =0.5$, then the decision-maker displays neutral performance, which demonstrates that the weight values of both benefit-type and cost-type attributes are equal.

**Step 5:** Based on the relative degree$\ \gamma _i$, the prioritization of each alternative is estimated. If the alternative with the highest relative degree$\ D^*$, it is the optimal alternative.

19 $$\begin{aligned} D^* = \left\{ D_i \vert \mathop {\max } \limits _i \gamma _i \right\} , \end{aligned}$$

where$$\ i=1,2,...,m$$.

**Step 6:** Rank the alternatives$\ P= \left\{ p_1,p_2,...,p_m \right\}$ based on the utility degree (UD)”. The larger the value of$\ UD(p_m)$, the better the preference ordering of alternative$\ p_i$, where$\ i=1,2,...,m$.

20 $$\begin{aligned} UD(p_i)=\frac{\gamma _i}{\mathop {\max }\nolimits _i \gamma _i}, \end{aligned}$$

where$$\ i=1,2,...,m$$.

### Example 2

Suppose that$\ p_1$ and$\ p_2$ are two alternatives, and$\ r_1$ and$\ r_2$ are two benefit attributes, and$\ \psi _1$ and$\ \psi _2$ are the IVFFNs weight of attributes$\ r_1$ and$\ r_2$ provided by the decision maker, respectively.

**[Step 1]** According to the decision maker’s opinion, the decision matrix$\ M=( \chi _{ij})_{2\times 2}=([\mu _{ij}^L,\mu _{ij}^U],[v_{ij}^L,v_{ij}^U])_{2\times 2}$ is as follows:where $1\leqslant i \leqslant 2$ and $1\leqslant j \leqslant 2$.

**[Step 2]** Based on the decision matrix $M=( \chi _{ij})_{2\times 2}=([\mu _{ij}^L,\mu _{ij}^U],[v_{ij}^L, v_{ij}^U])_{2\times 2}$, the weight of attributes can be obtained by using the CRITIC approach.

**[Step 2.1]** According to Eq. (5) and the decision matrix $M=( \chi _{ij})_{2\times 2} =([\mu _{ij}^L,\mu _{ij}^U],[v_{ij}^L,v_{ij}^U])_{2\times 2}$, the score matrix $SM =(\sigma _{ij})_{2\times 2}$ can be obtained, where $\sigma _{11}=0$, $\sigma _{12}=0$, $\sigma _{21}=0$ and $\sigma _{22}=0$. Then the score matrix $SM =(\sigma _{ij})_{2\times 2}$ is as follows:**[Step 2.2]** According to the score matrix$\ SM=(\sigma _{ij})_{2\times 2}$, build the normalized score matrix$\ {\tilde{SM}}=({\tilde{\sigma }}_{ij})_{2\times 2}$, where ${\tilde{\sigma }}_{11}=\frac{0}{0}$, $\ {\tilde{\sigma }}_{12}=\frac{0}{0}$, $\ {\tilde{\sigma }}_{21}=\frac{0}{0}$ and$\ {\tilde{\sigma }}_{22}=\frac{0}{0}$. Because$\ {\tilde{\sigma }}_{11}={\tilde{\sigma }}_{12}={\tilde{\sigma }}_{11}={\tilde{\sigma }}_{12}=\frac{0}{0}$, therefore, the remaining steps can not proceed. Due to the “division by zero” error, it is evident that Rani et al.’s MADM approach [52] cannot determine the preference orderings of alternatives in this circumstance.

In summary, when the interval values of the non-membership degree of the IVFFNs in the decision matrix are equal to the interval values of the membership degree, Rani’s MADM approach [52] fails to determine the preference orderings of the alternatives due to the division by zero error in the decision process. Therefore, Rani’s MADM approach [52] has the disadvantage that the preference orderings of alternatives cannot be derived in some situations.

## A new MADM approach based on the proposed score function of IVFFNs and the hybrid weighted score measure of IVFFNs

In this section, firstly, a novel score function is introduced in the interval-valued Fermatean environment. And then a new MADM approach is proposed based on the novel score function and the hybrid weighted score measure to address the disadvantage of Chen and Tsai’s MADM approach [54] and Rani et al.’s MADM approach [52].

### The proposed score function

Following is the new proposed SCF for IVFFNs.

#### Definition 10

Let $\tau =([\mu _t^L,\mu _t^U],[v_t^L,v_t^U])$ be an IVFFN, then the proposed SCF N of IVFFNs is defined as follows:

21 $$\begin{aligned} N(\tau )=\frac{{\mu _t^L}^3+{\mu _t^U}^3+{\mu _t^L}\root 3 \of {1-{v_t^L}^3}+{\mu _t^U}\root 3 \of {1-{v_t^U}^3}}{2} \end{aligned}$$

where $N(\tau )\in [0,2]$. The greater the magnitude of $N(\tau )$, then the greater the magnitude of the IVFFN $\tau$.

#### Property 1

Let $\alpha _1 =([\mu _1^L,\mu _1^U],[v_1^L,v_1^U])$ and $\alpha _2 =([\mu _2^L,\mu _2^U],[v_2^L,v_2^U])$ be two IVFFNs, If$\ \alpha _1>\alpha _2$, then $N(\alpha _1)>N(\alpha _2)$. If$\ \alpha _1<\alpha _2$, then $N(\alpha _1)<N(\alpha _2)$. If$\ \alpha _1=\alpha _2$, then $N(\alpha _1)=N(\alpha _2)$.

#### Proof

Based on Eq. (21), then$\ N(\alpha _1)=\frac{1}{2}\big ({\mu _1^L}^3+{\mu _1^U}^3+{\mu _1^L}\root 3 \of {1-{v_1^L}^3}+{\mu _1^U}\root 3 \of {1-{v_1^U}^3}\big )$ and$\ N(\alpha _2)=\frac{1}{2}\big ({\mu _2^L}^3+{\mu _2^U}^3+{\mu _2^L}\root 3 \of {1-{v_2^L}^3}+{\mu _2^U}\root 3 \of {1-{v_2^U}^3}\big )$.

$$\begin{aligned} \begin{aligned}&N(\alpha _1)-N(\alpha _2)\\&\quad =\frac{{\mu _1^L}^3+{\mu _1^U}^3+{\mu _1^L}\root 3 \of {1-{v_1^L}^3}+{\mu _1^U}\root 3 \of {1-{v_1^U}^3}}{2}\\&\qquad -\frac{{\mu _2^L}^3+{\mu _2^U}^3+{\mu _2^L}\root 3 \of {1-{v_2^L}^3}+{\mu _2^U}\root 3 \of {1-{v_2^U}^3}}{2}\\&\quad =\frac{1}{2}\bigg [ ({\mu _1^L}^3-{\mu _2^L}^3)+({\mu _1^U}^3-{\mu _2^U}^3) \\&\qquad +\big ({\mu _1^L}\root 3 \of {1-{v_1^L}^3}-{\mu _2^L}\root 3 \of {1-{v_2^L}^3}\big )\\&\qquad +\big ({\mu _1^U}\root 3 \of {1-{v_1^U}^3}-{\mu _2^U}\root 3 \of {1-{v_2^U}^3}\big ) \bigg ] \end{aligned} \end{aligned}$$

(i) If $\alpha _1>\alpha _2$, where $\alpha _1=([\mu _1^L,\mu _1^U], [v_1^L,v_1^U])$ and $\alpha _2=([\mu _2^L,\mu _2^U]$, $\ [v_2^L,v_2^U])$, then based on Definition 9, it is obvious that$\ \mu _1^L > \mu _2^L$, $\mu _1^U > \mu _2^U$, $v_1^L < v_2^L$ and$\ v_1^U < v_2^U$, then ${\mu _1^L}^3-{\mu _2^L}^3 > 0$, $\ {\mu _1^U}^3-{\mu _2^U}^3 > 0$. From Definition 2, there is$\ 0\leqslant \mu _1^L \leqslant \mu _1^U \leqslant 1$, $0\leqslant v_1^L \leqslant v_1^U \leqslant 1$, $0\leqslant \mu _2^L \leqslant \mu _2^U \leqslant 1$, $0\leqslant v_2^L \leqslant v_2^U \leqslant 1$. Because $\mu _1^L > \mu _2^L$, $\mu _1^U > \mu _2^U$, $v_1^L < v_2^L$ and $v_1^U < v_2^U$, then ${v_1^L}^3 < {v_2^L}^3$, ${v_1^U}^3 < {v_2^U}^3$. It can be seen that $1-{v_1^L}^3 > 1-{v_2^L}^3$, $\ 1-{v_1^U}^3 > 1-{v_2^U}^3$, $\root 3 \of {1-{v_1^L}^3} > \root 3 \of {1-{v_2^L}^3}$, $\root 3 \of {1-{v_1^U}^3} > \root 3 \of {1-{v_2^U}^3}$. Therefore,$\ {\mu _1^L}\root 3 \of {1-{v_1^L}^3}>{\mu _2^L}\root 3 \of {1-{v_2^L}^3}$, ${\mu _1^U}\root 3 \of {1-{v_1^U}^3}>{\mu _2^U}\root 3 \of {1-{v_2^U}^3}$, $\ {\mu _1^L}\root 3 \of {1-{v_1^L}^3}-{\mu _2^L}\root 3 \of {1-{v_2^L}^3} > 0$ and ${\mu _1^U}\root 3 \of {1-{v_1^U}^3}-{\mu _2^U}\root 3 \of {1-{v_2^U}^3} > 0$. Because
  $$\begin{aligned} \begin{aligned}&N(\alpha _1)-N(\alpha _2)\\&=\frac{{\mu _1^L}^3+{\mu _1^U}^3+{\mu _1^L}\root 3 \of {1-{v_1^L}^3}+{\mu _1^U}\root 3 \of {1-{v_1^U}^3}}{2}\\&-\frac{{\mu _2^L}^3+{\mu _2^U}^3+{\mu _2^L}\root 3 \of {1-{v_2^L}^3}+{\mu _2^U}\root 3 \of {1-{v_2^U}^3}}{2}, \end{aligned} \end{aligned}$$
  and because ${\mu _1^L}^3-{\mu _2^L}^3>0$, ${\mu _1^U}^3-{\mu _2^U}^3 >0$, ${\mu _1^L}\root 3 \of {1-{v_1^L}^3}-{\mu _2^L}\root 3 \of {1-{v_2^L}^3} > 0$ and $\ {\mu _1^U}\root 3 \of {1-{v_1^U}^3}-{\mu _2^U}\root 3 \of {1-{v_2^U}^3} > 0$, then $N(\alpha _1)-N(\alpha _2) > 0$. That is$\ N(\alpha _1)>N(\alpha _2)$. Therefore, if the IVFFNs$\ \alpha _1>\alpha _2$, then $N(\alpha _1)>N(\alpha _2)$.
(ii) If $\alpha _1<\alpha _2$, where $\alpha _1=([\mu _1^L,\mu _1^U], [v_1^L,v_1^U])$ and $\alpha _2=([\mu _2^L,\mu _2^U]$, $\ [v_2^L,v_2^U])$, then based on Definition 9, it is obvious that$\ \mu _1^L < \mu _2^L$, $\mu _1^U < \mu _2^U$, $v_1^L > v_2^L$ and$\ v_1^U > v_2^U$, then ${\mu _1^L}^3-{\mu _2^L}^3 < 0$, $\ {\mu _1^U}^3-{\mu _2^U}^3 < 0$. It can be seen from Definition 2, $0\leqslant \mu _1^L \leqslant \mu _1^U \leqslant 1$, $0\leqslant v_1^L \leqslant v_1^U \leqslant 1$, $0\leqslant \mu _2^L \leqslant \mu _2^U \leqslant 1$, $0\leqslant v_2^L \leqslant v_2^U \leqslant 1$. Because $\mu _1^L < \mu _2^L$, $\mu _1^U < \mu _2^U$, $v_1^L > v_2^L$ and $v_1^U > v_2^U$, then ${v_1^L}^3 > {v_2^L}^3$, ${v_1^U}^3 > {v_2^U}^3$. It can be seen that $1-{v_1^L}^3 < 1-{v_2^L}^3$, $\ 1-{v_1^U}^3 < 1-{v_2^U}^3$, $\root 3 \of {1-{v_1^L}^3} < \root 3 \of {1-{v_2^L}^3}$, $\root 3 \of {1-{v_1^U}^3} < \root 3 \of {1-{v_2^U}^3}$. Therefore,$\ {\mu _1^L}\root 3 \of {1-{v_1^L}^3}<{\mu _2^L}\root 3 \of {1-{v_2^L}^3}$, ${\mu _1^U}\root 3 \of {1-{v_1^U}^3}<{\mu _2^U}\root 3 \of {1-{v_2^U}^3}$, $\ {\mu _1^L}\root 3 \of {1-{v_1^L}^3}-{\mu _2^L}\root 3 \of {1-{v_2^L}^3} < 0$ and ${\mu _1^U}\root 3 \of {1-{v_1^U}^3}-{\mu _2^U}\root 3 \of {1-{v_2^U}^3} < 0$. Because
  $$\begin{aligned} \begin{aligned}&N(\alpha _1)-N(\alpha _2)\\&=\frac{{\mu _1^L}^3+{\mu _1^U}^3+{\mu _1^L}\root 3 \of {1-{v_1^L}^3}+{\mu _1^U}\root 3 \of {1-{v_1^U}^3}}{2}\\&-\frac{{\mu _2^L}^3+{\mu _2^U}^3+{\mu _2^L}\root 3 \of {1-{v_2^L}^3}+{\mu _2^U}\root 3 \of {1-{v_2^U}^3}}{2}, \end{aligned} \end{aligned}$$
  and because ${\mu _1^L}^3-{\mu _2^L}^3<0$, $\ {\mu _1^U}^3-{\mu _2^U}^3<0$, $\ {\mu _1^L}\root 3 \of {1-{v_1^L}^3}-{\mu _2^L}\root 3 \of {1-{v_2^L}^3}< 0$ and$\ {\mu _1^U}\root 3 \of {1-{v_1^U}^3}-{\mu _2^U}\root 3 \of {1-{v_2^U}^3} < 0$, then $N(\alpha _1)-N(\alpha _2) < 0$. That is$\ N(\alpha _1)<N(\alpha _2)$. Therefore, if the IVFFNs$\ \alpha _1<\alpha _2$, then $N(\alpha _1)<N(\alpha _2)$.
(iii) If $\alpha _1=\alpha _2$, where $\alpha _1=([\mu _1^L,\mu _1^U], [v_1^L,v_1^U])$ and $\alpha _2=([\mu _2^L,\mu _2^U]$, $\ [v_2^L,v_2^U])$, then based on Definition 9, it is obvious that$\ \mu _1^L = \mu _2^L$, $\mu _1^U = \mu _2^U$, $v_1^L = v_2^L$ and$\ v_1^U = v_2^U$, then ${\mu _1^L}^3-{\mu _2^L}^3 = 0$, $\ {\mu _1^U}^3-{\mu _2^U}^3 = 0$. It can be seen from Definition 2, $0\leqslant \mu _1^L \leqslant \mu _1^U \leqslant 1$, $0\leqslant v_1^L \leqslant v_1^U \leqslant 1$, $0\leqslant \mu _2^L \leqslant \mu _2^U \leqslant 1$, $0\leqslant v_2^L \leqslant v_2^U \leqslant 1$. Because $\mu _1^L = \mu _2^L$, $\mu _1^U = \mu _2^U$, $v_1^L = v_2^L$ and $v_1^U = v_2^U$, then ${v_1^L}^3 = {v_2^L}^3$, ${v_1^U}^3 = {v_2^U}^3$. It can be seen that$\ 1-{v_1^L}^3 = 1-{v_2^L}^3$, $1-{v_1^U}^3 = 1-{v_2^U}^3$, $\ \root 3 \of {1-{v_1^L}^3} = \root 3 \of {1-{v_2^L}^3}$, $\ \root 3 \of {1-{v_1^U}^3} = \root 3 \of {1-{v_2^U}^3}$. Therefore,$\ {\mu _1^L}\root 3 \of {1-{v_1^L}^3}={\mu _2^L}\root 3 \of {1-{v_2^L}^3}$, $\ {\mu _1^U}\root 3 \of {1-{v_1^U}^3}={\mu _2^U}\root 3 \of {1-{v_2^U}^3}$, $\ {\mu _1^L}\root 3 \of {1-{v_1^L}^3}-{\mu _2^L}\root 3 \of {1-{v_2^L}^3} = 0$ and ${\mu _1^U}\root 3 \of {1-{v_1^U}^3}-{\mu _2^U}\root 3 \of {1-{v_2^U}^3} = 0$. Because
  $$\begin{aligned} \begin{aligned}&N(\alpha _1)-N(\alpha _2)\\&=\frac{{\mu _1^L}^3+{\mu _1^U}^3+{\mu _1^L}\root 3 \of {1-{v_1^L}^3}+{\mu _1^U}\root 3 \of {1-{v_1^U}^3}}{2}\\&-\frac{{\mu _2^L}^3+{\mu _2^U}^3+{\mu _2^L}\root 3 \of {1-{v_2^L}^3}+{\mu _2^U}\root 3 \of {1-{v_2^U}^3}}{2}, \end{aligned} \end{aligned}$$
  and because ${\mu _1^L}^3-{\mu _2^L}^3=0$, $\ {\mu _1^U}^3-{\mu _2^U}^3=0$, $\ {\mu _1^L}\root 3 \of {1-{v_1^L}^3}-{\mu _2^L}\root 3 \of {1-{v_2^L}^3} = 0$ and$\ {\mu _1^U}\root 3 \of {1-{v_1^U}^3}-{\mu _2^U}\root 3 \of {1-{v_2^U}^3} = 0$, then $N(\alpha _1)-N(\alpha _2) = 0$. That is $N(\alpha _1)=N(\alpha _2)$. Therefore, if the IVFFNs $\alpha _1=\alpha _2$, then $N(\alpha _1)=N(\alpha _2)$. $\square$

#### Property 2

Let *t* be an IVFFN, where $\tau =([\mu _t^L,\mu _t^U], [v_t^L,v_t^U])$, where $0 \leqslant \mu _t^L \leqslant \mu _t^U \leqslant 1$, $0 \leqslant v_t^L \leqslant v_t^U \leqslant 1$, and $0 \leqslant {\mu _t^U}^3+{v_t^U}^3 \leqslant 1$. Then,$\ N(\tau ) \in [0,2]$.

#### Proof

Because $0 \leqslant \mu _t^L \leqslant \mu _t^U \leqslant 1$ and $0 \leqslant v_t^L \leqslant v_t^U \leqslant 1$, then $0 \leqslant {\mu _t^L}^3 \leqslant {\mu _t^U}^3 \leqslant 1$, $0 \leqslant {v_t^L}^3 \leqslant {v_t^U}^3 \leqslant 1$, where ${\mu _t^L}^3, {\mu _t^U}^3, {v_t^L}^3, {v_t^U}^3 \in [0,1]$, $1-{v_t^L}^3 \in [0,1]$, $1-{v_t^U}^3 \in [0,1]$. From Eq. (21),$\ N(\tau )=({\mu _t^L}^3+{\mu _t^U}^3+{\mu _t^L}\root 3 \of {1-{v_t^L}^3}+{\mu _t^U}\root 3 \of {1-{v_t^U}^3})/2$, where $\tau =([\mu _t^L,\mu _t^U],[v_t^L,v_t^U])$. Because$\ {\mu _t^L}^3, {\mu _t^U}^3, {v_t^L}^3, {v_t^U}^3 \in [0,1]$, $\ 1-{v_t^L}^3 \in [0,1]$, $1-{v_t^U}^3 \in [0,1]$, then $N(\tau ) \in [0,2]$. $\square$

#### Property 3

If the IVFFN $\tau =([0,0],[1,1])$, then $N(\tau ) = 0$.

#### Proof

Because the IVFFN $\tau =([0,0],[1,1])$, based on Eq. (21),

$$\begin{aligned} \begin{aligned} N(\tau ) = \frac{ {0}^3+0^3+0\root 3 \of {1-1^3}+0\root 3 \of {1-1^3}}{2}= \frac{0}{2} = 0. \end{aligned} \end{aligned}$$

$$\square $$

#### Property 4

If the IVFFN $\tau =([1,1],[0,0])$, then $N(\tau ) = 2$.

#### Proof

Because the IVFFN $\tau =([1,1],[0,0])$, based on Eq. (21), then

$$\begin{aligned} \begin{aligned} N(\tau ) = \frac{{1}^3+1^3+1\root 3 \of {1-0^3}+1\root 3 \of {1-0^3}}{2}= \frac{4}{2} = 2. \end{aligned} \end{aligned}$$

$$\square $$

### The proposed MADM approach

**Fig. 1:**
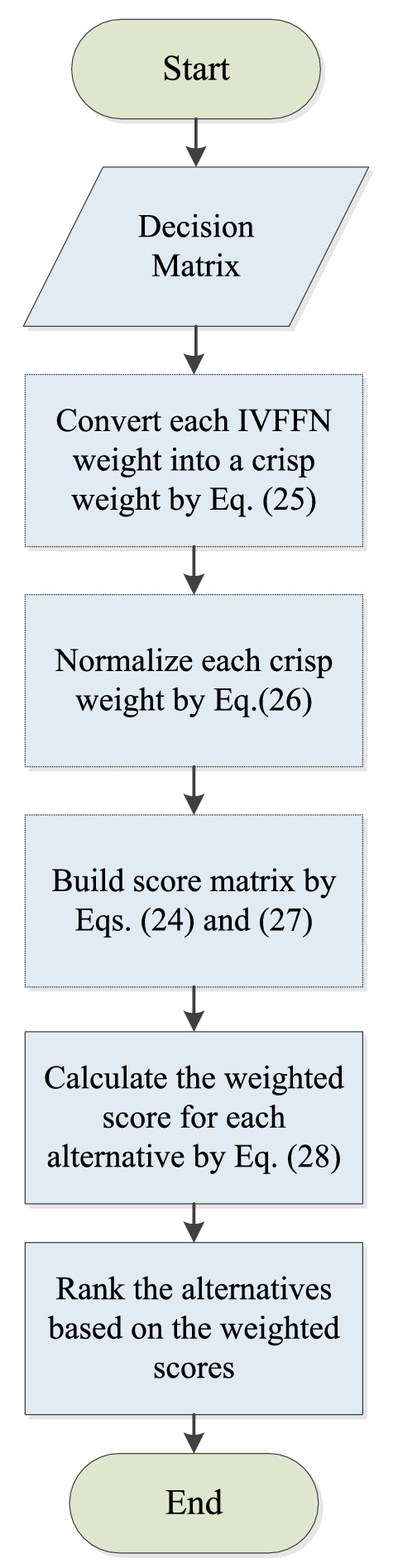
The proposed MADM approach flow chart

Let $r_1,r_2,...,$ and $r_n$ be attributes, let $p_1,p_2,...,$ and $p_m$ be alternatives, and let decision matrix $M=( \chi _{ij})_{m\times n}=([\mu _{ij}^L,\mu _{ij}^U],[v_{ij}^L,v_{ij}^U])_{m\times n} (i=1,2,...,m, j=1,2,...,n)$, where $[\mu _{ij}^L,\mu _{ij}^U]\in [0,1], [v_{ij}^L,v_{ij}^U]\in [0,1]$, and $0 \leqslant {\mu _{ij}^U}^3+{v_{ij}^U}^3 \leqslant 1$. Assume that$\ \varPsi =(\psi _1,\psi _2,...,\psi _n)^T$ be the group of attribute weight with $\psi _j=([\varepsilon _j^L,\varepsilon _j^U],[\epsilon _j^L,\epsilon _j^U])$. Figure 1 shows the process for developing the MADM approach. In addition, its steps of implementation are following.

**Step 1:** Based on our proposed SCF shown in Eq. (21), convert each IVFFN weight$\ \psi _j=([\varepsilon _j^L,\varepsilon _j^U],[\epsilon _j^L,\epsilon _j^U])$ into a crisp weight ${\tilde{\psi }}_j$, where

22 $$\begin{aligned} {\tilde{\psi }}_j=\frac{{\varepsilon _j^L}^3+{\varepsilon _j^U}^3+{\varepsilon _j^L}\root 3 \of {1-{\epsilon _j^L}^3}+{\varepsilon _j^U}\root 3 \of {1-{\epsilon _j^U}^3}}{2}, \end{aligned}$$

$${\tilde{\psi }}_j \in [0,2]$$ and $$1 \leqslant j \leqslant n$$.

**Step 2:** Obtain the normalized weight ${\bar{\psi }}_j$ for attribute $r_j$, as shown below:

23 $$\begin{aligned} {\bar{\psi }}_j=\frac{{\tilde{\psi }}_j}{\sum _{j=1}^{n}{\tilde{\psi }}_j}, \end{aligned}$$

where $${\bar{\psi }}_j \in [0,1]$$, $$ 1 \leqslant j \leqslant n$$ and$$\ \sum _{j=1}^{n} {\bar{\psi }}=1$$.

**Step 3:** Based on our proposed SCF shown in Eq. (21) and the decision matrix $M=( \chi _{ij})_{m\times n} =([\mu _{ij}^L,\mu _{ij}^U], [v_{ij}^L,v_{ij}^U])_{m\times n}$, build the score matrix $SM =(\sigma _{ij})_{m\times n}$, where

24 $$\begin{aligned} \sigma _{ij}= \left\{ \begin{matrix} N(\chi _{ij}), \quad r_j\in R_1\\ 1-N(\chi _{ij}), \quad r_j \in R_2 \end{matrix}\right. , \end{aligned}$$

and $R_1$ is the set of benefit attributes and $R_2$ is the set of cost attributes, and$\ N(\chi _{ij})=\big ({\mu _{ij}^L}^3+{\mu _{ij}^U}^3+{\mu _{ij}^L}\root 3 \of {1-{v_{ij}^L}^3}+{\mu _{ij}^U}\root 3 \of {1-{v_{ij}^U}^3}\big )/2$, $\ N(\chi _{ij}) \in [0,2]$, $1 \leqslant i \leqslant m$ and $1 \leqslant j \leqslant n$.

**Step 4:** According to the normalized weight$\ {\tilde{\psi }}_j$ and the score matrix $SM =(\sigma _{ij})_{m\times n}$, mix the “Weighted Sum Model (WSM)” and the “Weighted Product Model (WPM)” to form the “Hybrid Weighted Score Model (HWM)”, and then use HWM to calculate the weighted score $\eta _i$ for each alternative $p_i$.

25 $$\begin{aligned} \eta _i=\theta \sum _{j=1}^{n}{\tilde{\psi }_j}\sigma _{ij}+(1-\theta )\prod _{j=1}^{n}{\tilde{\psi }_j}\sigma _{ij}, \end{aligned}$$

where the parameter $\theta$ represents the coefficient of decision-making, such that $\theta \in [0,1]$ (when $\theta =0$, HWM is transformed into WPM, and when $\theta =1$, HWM is transformed into WSM).

**Step 5:** Rank the alternatives $P= \left\{ p_1,p_2,...,p_m \right\}$ based on the weighted scores $\eta _1, \eta _2, ...$, and $\eta _m$. The greater the value of $\eta _i$, the superior the preference ordering of alternative $p_i$, where$\ i=1,2,...,m$.

Furthermore, according to the proposed MADM approach process, computational complexity is given. In detail, the time complexity for converting each IVFFN weight into a crisp weight and normalizing each crisp weight is O(n). The time complexity for building the score matrix and calculating the weighted score for each alternative is O(mn). The last step’s time complexity for ranking the alternatives is $O(m{log_2}^m)$. Therefore, the proposed MADM approach’s time complexity is $O(mn)+O(n)+O(m{log_2}^m)$.

### Illustrate examples

#### Example 3

Using the same premises as in Example 1, where $p_1$ and $p_2$ are two alternatives, $\psi _1$ = ([0.20,0.35], [0.10,0.50]) and $\psi _2=([0.20,0.25],[0.30,0.40])$ are the IVFFNs weight of benefit-type attributes $r_1$ and $r_2$, respectively. And the decision matrix $M=( \chi _{ij})_{2\times 2} =([\mu _{ij}^L,\mu _{ij}^U],[v_{ij}^L,v_{ij}^U])_{2\times 2}$ is shown as follows:where $1\leqslant i \leqslant 2$ and $1\leqslant j \leqslant 2$.

Next, using our proposed approach to deal with the problem in Example 1, as shown in the following steps.

**[Step 1]** Based on our proposed SCF shown in Eq. (21), convert the IVFFNs weight $\psi _1=([0.20,0.35], [0.10,0.50])$ and $\psi _2=([0.20,0.25],[0.30, 0.40])$ of attributes $r_1$ and $r_2$ into the crisp weight ${\tilde{\psi }}_1$ and$\ {\tilde{\psi }}_2$, where ${\tilde{\psi }}_1=0.2928$ and$\ {\tilde{\psi }}_2=0.2332$.

**[Step 2]** According to the crisp weight ${\tilde{\psi }}_1$ and ${\tilde{\psi }}_2$, then the normalized weight ${\bar{\psi }}_1=0.5567$ and ${\bar{\psi }}_2=0.4433$ for attribute $r_1$ and $r_2$, respectively.

**[Step 3]** Based on our introduced SCF shown in Eq. (21) and the decision matrix $M=( \chi _{ij})_{2\times 2} =([\mu _{ij}^L,\mu _{ij}^U],[v_{ij}^L,v_{ij}^U])_{2\times 2}$ to build the score matrix $SM =(\sigma _{ij})_{2\times 2}$, and because $r_1$ and $r_2$ are benefit attributes. Then, the score matrix $SM =(\sigma _{ij})_{2\times 2}$ can be obtained as follows, where $\sigma _{11}=0.1258$, $\sigma _{12}=0.0200$, $\ \sigma _{21}=0.0981$ and $\sigma _{22}=0.0100$.**[Step 4]** According to the normalized weight$\ {\bar{\psi }}_1=0.5567$ and ${\bar{\psi }}_2=0.4433$, and the score matrix $SM =(\sigma _{ij})_{2\times 2}$, the weighted score$\ \eta _1$ and $\eta _2$ for each alternative $p_1$ and $p_2$ can be derived by using HWM. For convenience, the coefficient parameter is set to 0.5. Therefore, the weighted score of alternatives $p_1$ and $p_2$ can be obtained, where $\eta _1=0.5(0.1258 \times 0.5567+0.0200 \times 0.4433)+0.5(0.1258 \times 0.5567 \times 0.0200 \times 0.4433)=0.0398$ and $\eta _2=0.5(0.0981 \times 0.5667+0.0100 \times 0.4433)+0.5(0.0981 \times 0.5667 \times 0.0100 \times 0.4433)=0.0301$.

**[Step 5]** Rank the alternatives based on the above weighted score. Because $\eta _1>\eta _2$, the preference orderings between alternatives $p_1$ and $p_2$ is “$p_1>p_2$”.

Therefore, our proposed MADM approach can overcome the drawback that Chen and Tsai’s MADM approach [54] cannot distinguish the preference orderings of alternatives $p_1$ and$\ p_2$ in this situation, as shown in Example 1.

#### Example 4

Consider the same decision matrix shown in Example 2, $p_1$ and $p_2$ are two alternatives, $\psi _1 =([0.21,0.45],[0.12,0.40])$ and $\psi _2=$ ([0.14,0.26], [0.29,0.40]) are the IVFFNs weight of benefit-type attributes $r_1$ and $r_2$, respectively. And the decision matrix $M=( \chi _{ij})_{2\times 2} =([\mu _{ij}^L,\mu _{ij}^U],[v_{ij}^L,v_{ij}^U])_{2\times 2}$ is shown as follows:where $1\leqslant i \leqslant 2$ and $1\leqslant j \leqslant 2$.

Our MADM method is applied to cope with the data in Example 2 as follows.

**[Step 1]** Transform the IVFFNs weight $\psi _1=$ ([0.21,0.45], [0.12,0.40]) and $\psi _2=$ ([0.14,0.26], [0.290, 0.40]) of attributes $r_1$ and $r_2$ into the crisp weight ${\tilde{\psi }}_1$ and ${\tilde{\psi }}_2$ by our proposed SCF. As a result, ${\tilde{\psi }}_1=0.3752$ and ${\tilde{\psi }}_2=0.2068$.

**[Step 2]** Obtain the normalized weight according to the above crisp weight as ${\bar{\psi }}_1=0.6447$ and$\ {\bar{\psi }}_2=0.3553$ for attribute $r_1$ and $r_2$, respectively.

**[Step 3]** Build the score matrix $SM =(\sigma _{ij})_{2\times 2}$ shown as follows, by means of our proposed SCF and the above decision matrix (Here, $r_1$ and $r_2$ are benefit attributes).**[Step 4]** Compute the weighted score $\eta _1$ and $\eta _2$ for alternatives $p_1$ and $p_2$ by Eq. (25), according to the normalized weight and the score matrix$\ SM=(\sigma _{ij})_{2\times 2}$. The coefficient parameter $\theta$ is given as 0.5. Therefore, the weighted score of alternatives$\ p_1$ and $p_2$ can be derived, where $\eta _1=0.1581$ and$\ \eta _2=0.1781$.

**[Step 5]** Sort the alternatives $p_1$ and $p_2$ according to the weighted score $\eta _1=0.1581$ and $\eta _2=0.1781$. Since$\ \eta _1<\eta _2$, it is easily found that the preference orderings between alternatives $p_1$ and $p_2$ is “$p_1<p_2$”.

Therefore, our new MADM method outperforms the approach in [52] in this situation. In addition, our MADM approach eliminates the possibility of not obtaining the preference orderings of the alternatives due to zero division during the operation, as shown in Example 2.

From the validations of the above two examples, the preference orderings of alternatives can be derived from our proposed MADM approach in any situation. In contrast to the MADM methods in Rani et al. [52] and Chen and Tsai [54]. It can be seen from Example 4, when MSD and NMSD have the same interval value, there is a division by 0 error in Rani et al.’s MADM process [52].

## Applications of the proposed MADM method

To illustrate the proposed method’s utility and superiority, three real-life applications are applied to demonstrate the proposed approach for solving MADM problems.

**Table 1:** The interval-valued Fermatean fuzzy CEDI systems decision matrix *M* for case 1

	$r_1$	$r_2$	$r_3$	$r_4$
$p_1$	([0.55,0.74], [0.03,0.04])	([0.44,0.59], [0.12,0.17])	([0.55,0.74], [0.03,0.04])	([0.24,0.33], [0.27,0.38])
$p_2$	([0.51,0.70], [0.04,0.06])	([0.42,0.58], [0.12,0.16])	(([0.44,0.60], [0.12,0.16])	([0.13,0.18], [0.43,0.58])
$p_3$	([0.51,0.69], [0.04,0.06])	([0.48,0.66], [0.04,0.06])	([0.38,0.54], [0.10,0.14])	([0.06,0.08], [0.50,0.67])
$p_4$	([0.55,0.74], [0.03,0.04])	([0.48,0.66], [0.05,0.07])	([0.39,0.56], [0.08,0.11])	([0.12,0.16], [0.42,0.57])
$p_5$	([0.54,0.73], [0.02,0.03])	([0.41,0.57], [0.11,0.16])	([0.41,0.58], [0.08,0.11])	([0.22,0.30], [0.29,0.41])

**Case 1.** To demonstrate the utility of the proposed approach, it was applied to the evaluation of clean energy-driven desalination-irrigation (CEDI) systems in the Xinjiang Uygur Autonomous Region of Northwest China(modified from [56]). The Xinjiang Uygur Autonomous Region is the largest administrative division in China, with an area of more than 1.6 million square kilometers, accounting for about one-sixth of China’s land area. Due to the distance from the ocean, the region forms a typical temperate continental arid climate, with an average annual precipitation of only 163.3 mm. Xinjiang is a region that is highly dependent on irrigation due to low rainfall. Although Xinjiang lacks freshwater resources, its per capita water resources are only 3130 cubic meters, and Xinjiang has considerable potential for brackish water resources [56].

In order to select the optimal CEDI system from five different CEDI systems $p_i(i=1,2,...,3)$, expert selected four attributes to evaluate the systems. Three attributes $r_j(j=1,2,...,4)$ relevant to the economy, agriculture, environment, and system performance were selected by consulting experts, which are $r_1$: product yield, $r_2$: system stability, $r_3$: system adaptability, and$\ r_4$: integrated environmental impact. In this paper, product yield ($r_1$), system stability ($r_2$), and system adaptability ($r_3$) are benefit attributes. The higher stability of the system, the better the product yield, and the better adaptability of the system, which means the better performance of the CEDI system, while the rest of the attribute is considered cost attribute. Let$\ \psi _1=([0.25,0.30],[0.15,0.20])$, $\psi _2=([0.30,0.40],\ [0.10,0.15])$, $\psi _3=([0.40,0.45],[0.20,0.25])$ and$\ \psi _4=([0.05,0.10],[0.30,0.35])$ be the weight of each attribute. This paper collects experts’ evaluation opinions on five different CEDI systems and obtains the decision matrix $M=( \chi _{ij})_{5\times 4}=([\mu _{ij}^L,\mu _{ij}^U],[v_{ij}^L, v_{ij}^U])_{5\times 4}$ shown as Table 1.

**Input:** Decision matrix $M=( \chi _{ij})_{5\times 4}$ for the evaluation of the candidates.

**Step 1:** According to our proposed SCF, convert the IVFFNs weight $\psi _1=([0.25,0.30],[0.15,0.20])$, $\psi _2=([0.30,0.40],[0.10,0.15])$, $\psi _3=([0.40,0.45],[0.20,0.25])$ and $\psi _4=([0.05,0.10],[0.30,0.35])$ of attributes $r_1$, $r_2$, $r_3$, and $r_4$ into the crisp weight ${\tilde{\psi }}_1$, ${\tilde{\psi }}_2$ and ${\tilde{\psi }}_3$, where ${\tilde{\psi }}_1=0.2958$, ${\tilde{\psi }}_2=0.3952$, ${\tilde{\psi }}_3=0.5008$ and ${\tilde{\psi }}_4=0.0746$.

**Step 2:** Based on the above crisp weights, then the normalized weight ${\bar{\psi }}_1=0.2336$, ${\bar{\psi }}_2=0.3121$, $\ {\bar{\psi }}_3=0.3955$ and ${\bar{\psi }}_4=0.0589$ for attribute$\ r_1$, $r_2$, $r_3$ and $r_4$, respectively.

**Step 3:** Create the score matrix $SM =(\sigma _{ij})_{5\times 4}$ based on our proposed SCF and the decision matrix (Here, $r_1$, $r_2$ and $r_3$ are benefit attributes). Therefore, the score matrix $SM =(\sigma _{ij})_{5\times 4}$ can be derived as follows.**Step 4:** Apply HWM to calculate the weighted score$\ \eta _1$, $\eta _2$, $\eta _3$ and $\eta _4$ for each alternative$\ p_1$, $p_2$, $p_3$, $p_4$ and $p_5$ according to the above normalized weight. Here, the coefficient parameter is $\ \theta =0.5$ into account. Therefore, the weighted score of alternatives $p_1$, $p_2$, $p_3$, $p_4$ and $p_5$ can be obtained, where $\eta _1=0.4165$, $\eta _2=0.3552$, $\ \eta _3=0.3567$, $\eta _4=0.3716$ and $\eta _5=0.3486$.

**Step 5:** Rank the alternatives based on the above weighted score. Because $\eta _1>\eta _4>\eta _3>\eta _2>\eta _5$, then “$p_1>p_4>p_3>p_2>p_5$”.

**Output:** The preference orderings of the candidates is$\ p_1>p_4>p_3>p_2>p_5$.

In Case 1, the preference orderings of the alternatives obtained by Chen and Tsai’s MADM approach [54] is$\ p_1>p_4>p_3>p_2>p_5$. Besides, the preference orderings of the alternatives derived by Rani et al.’s MADM approach [52] is also $p_1>p_4>p_3>p_2>p_5$. Obviously, our proposed approach is reasonable and practical.

**Case 2.** A university wants to hire a talented faculty member, which is a problem in the area of human resource management (modified from [57]). The university convenes a group of decision-makers. After carefully analyzing the information, they critically assess three candidates$\ p_i(i=1,2,...,3)$ in these three main dimensions$\ r_j(j=1,2,3)$, where $r_1$: research skills, $r_2$: teaching skills, and $r_3$: educational background. Let$\ \psi _1=([0.15,0.30],[0.15,0.45])$, $\ \psi _2=([0.10,0.20],[0.35,0.45])$ and$\ \psi _3=([0.25,0.30],[0.15,0.20])$ be the weight of each attribute. The evaluation information of the decision makers on the candidates is used to obtain the decision matrix ${\bar{M}}=( \chi _{ij})_{3\times 3}=([\mu _{ij}^L,\mu _{ij}^U],[v_{ij}^L, v_{ij}^U])_{3\times 3}$ is shown as Table 2.

**Table 2:** The interval-valued Fermatean fuzzy human resource decision matrix ${\bar{M}}$ for case 2

	$r_1$	$r_2$	$r_3$
$p_1$	([0.01, 0.04], [0.19, 0.75])	([0.16, 0.25], [0.19, 0.51])	([0.09, 0.16], [0.51, 0.75])
$p_2$	([0.09, 0.09], [0.51, 0.84])	([0.16, 0.36], [0.36, 0.51])	([0.01, 0.09], [0.36, 0.51])
$p_3$	([0.01, 0.04],[0.36, 0.64])	([0.04, 0.25],[0.19, 0.19])	([0.00, 0.04], [0.19, 0.36])

**Table 3:** The interval-valued Fermatean fuzzy invest decision matrix $\breve{M}$ for case 3

	$r_1$	$r_2$	$r_3$
$p_1$	([0.24, 0.48], [0.24, 0.48])	([0.45, 0.47], [0.45, 0.47])	([0.32, 0.40], [0.32, 0.40])
$p_2$	([0.25, 0.36], [0.25, 0.36])	([0.16, 0.21], [0.16, 0.21])	([0.06, 0.14], [0.06, 0.14])
$p_3$	([0.34, 0.47], [0.34, 0.47])	([0.21, 0.38], [0.21, 0.38])	([0.18, 0.20], [0.18, 0.20])

**Input:** Decision matrix ${\bar{M}}=( \chi _{ij})_{3\times 3}$ for the evaluation of the candidates.

**Step 1:** According to our proposed SCF, convert the IVFFNs weight $\psi _1=([0.15,0.30],[0.15,0.45])$, $\ \psi _2=([0.10,0.20],[0.35,0.45])$ and$\ \psi _3=([0.25,0.30],[0.15,0.20])$ of attributes $r_1$, $r_2$ and$\ r_3$ into the crisp weight ${\tilde{\psi }}_1$, ${\tilde{\psi }}_2$ and ${\tilde{\psi }}_3$, where ${\tilde{\psi }}_1=0.2354$, $\ {\tilde{\psi }}_2=0.1506$ and ${\tilde{\psi }}_3=0.2958$.

**Step 2:** Based on the above crisp weights, then the normalized weight ${\bar{\psi }}_1=0.3453$, ${\bar{\psi }}_2=0.2209$ and ${\bar{\psi }}_3=0.4339$ for attribute $r_1$, $r_2$ and $r_3$, respectively.

**Step 3:** Create the score matrix $SM =(\sigma _{ij})_{3\times 3}$ based on our proposed SCF and the decision matrix (Here, $r_1$, $r_2$ and $r_3$ are benefit attributes). Therefore, the score matrix $SM =(\sigma _{ij})_{3\times 3}$ can be obtained as follows.**Step 4:** Apply HWM to calculate the weighted score$\ \eta _1$, $\eta _2$ and $\eta _3$ for each alternative $p_1$, $p_2$ and $p_3$ according to the above normalized weight. Here, the coefficient parameter is $\ \theta =0.5$. Therefore, the weighted score of alternatives $p_1$, $p_2$ and $p_3$ can be derived, where $\eta _1=0.0511$, $\eta _2=0.0542$ and $\eta _3=0.0251$.

**Step 5:** Rank the alternatives based on the above weighted score. Because $\eta _2>\eta _1>\eta _3$, then “$p_2>p_1>p_3$”.

**Output:** The preference orderings of the candidates is$\ p_2>p_1>p_3$.

However, the preference orderings of the alternatives derived using the MADM method proposed by Chen and Tsai [54] is $p_1=p_2=p_3$. Therefore, Chen and Tsai’s MADM method [54] cannot derive the preference orderings of the alternatives in Case 2.

**Case 3.** A fund manager of a wealth management company desires to make an investment decision on three potential investment opportunities(modified from [58]), denoted as $p_1$, $p_2$ and $p_3$, the fund manager is authorized to evaluate each investment based on three attributes$\ r_j(j=1,2,3)$, where $r_1$: risk, $r_2$: growth and $r_3$: environmental impact. Let $\psi _1=$ ([0.25, 0.32], [0.10, 0.21]), $\psi _2=$ ([0.16,0.24], [0.34,0.38]) and $\psi _3=$ ([0.37,0.40], [0.25,0.30]) be the weight of each attribute. The fund manager’s evaluation of the alternatives’ investment attractiveness, resulting in an investment decision matrix$\ \breve{M}=( \chi _{ij})_{3\times 3}=([\mu _{ij}^L,\mu _{ij}^U],[v_{ij}^L,v_{ij}^U])_{3\times 3}$ is shown as Table 3.

**Input:** Decision matrix $\breve{M}=( \chi _{ij})_{3\times 3}$ for the evaluation of the investment opportunities.

**Step 1:** Convert the above IVFFNs weights of attributes$\ r_1$, $r_2$ and $r_3$ into the crisp weights. As a result,$\ {\tilde{\psi }}_1=0.3087$, ${\tilde{\psi }}_2=0.2057$ and$\ {\tilde{\psi }}_3=0.4395$.

**Step 2:** Caculate the normalized weights by Eq. (23) as ${\bar{\psi }}_1=0.3236$, ${\bar{\psi }}_2=0.2156$ and$\ {\bar{\psi }}_3=0.4607$ for attribute $r_1$, $r_2$ and $r_3$, respectively.

**Step 3:** Achieve the score matrix $SM =(\sigma _{ij})_{3\times 3}$ according to our SCF and the above decision matrix. Here, $r_1$, $r_2$ and $r_3$ are considered as benefit attributes. Therefore, the score matrix $SM =(\sigma _{ij})_{3\times 3}$ can be obtained as follows.**Step 4:** Get the weighted score $\eta _1$, $\eta _2$ and$\ \eta _3$ for alternatives by means of HWM, according to the normalized weights and the score matrix $SM =(\sigma _{ij})_{3\times 3}$. The coefficient parameter $\theta$ is given as 0.5. Therefore, the weighted score of alternatives $p_1$, $p_2$ and $p_3$ can be derived, where $\eta _1=0.0528$, $\eta _2=0.0097$ and$\ \eta _3=0.0251$.

**Step 5:** Rank the alternatives according to the weighted score $\eta _1=0.2193$, $\eta _2=0.0979$ and $\eta _3=0.1520$. It is clear that $\eta _1>\eta _3>\eta _2$. Thus the preference orderings among alternatives $p_1$, $p_2$ and $p_3$ is “$p_1>p_3>p_2$”.

**Output:** The preference orderings of the three potential investment opportunities is $p_2>p_1>p_3$.

However, the preference orderings of the alternatives cannot derive using Rani et al.’s MADM method [52] in Case 3 because of the error of dividing by 0 in solving the MADM problem in Case 3.

## Comparison with the two existing MADM approaches

In this section, firstly, we compare our proposed score function with the six existing score functions to compare the differentiation rate. And then we compare our method with existing MADM methods to demonstrate the advantages of our proposed method: (1) our proposed method has the highest recognition index compared with existing methods; (2) our proposed MADM method can avoid the division by zero error when the interval values of the non-membership degrees of the IVFFNs in the decision matrix are equal to the interval values of the membership degrees.

### The proposed SCF vs. six existing SCFs

Next, analyze score functions in Rani and Mishra [53], Jeevaraj [25], Rani et al. [52] and Chen and Tsai [54] in some situations that cannot distinguish between any two IVFFNs. In addition, the examples demonstrate that our suggested new SCF overcomes the above shortcomings.

#### Example 5

Let $\alpha _1 =([\root 3 \of {0.40},\root 3 \of {0.60}],[\root 3 \of {0.15},\root 3 \of {0.20}])$ and $\alpha _2 =([\root 3 \of {0.35},\root 3 \of {0.45}],[\root 3 \of {0.05},\root 3 \of {0.10}])$ be two IVF-FNs. Then there is

1. Based on the SCF of Rani and Mishra [53] of IVFFNs shown in Eq. (1), then it obtains$\ M(\alpha _1)=M(\alpha _2)=0.325$, but $\alpha _1$ and $\alpha _2$ are two different IVFFNs. It is clear that Rani and Mishra’s SCF [53] cannot distinguish between any two IVFFNs. In other words, Rani and Mishra’s SCF [53] has the shortcoming that it cannot identify the preference ordering of IVFFNs $\alpha _1$ and $\alpha _2$ in this situation.
2. Based on our proposed new SCF shown in Eq. (21), $N(\alpha _1)=1.241$ and $N(\alpha _2)=1.116$. Because $N(\alpha _1)=1.241 > N(\alpha _2)=1.116$, the preference ordering between the IVFFNs $\alpha _1$ and $\alpha _2$ acquired by our proposed new SCF is “$\alpha _1 > \alpha _2$”.

#### Example 6

Let $\alpha _3 =([\root 3 \of {0.20},\root 3 \of {0.40}],[\root 3 \of {0.15},\root 3 \of {0.35}])$ and $\alpha _4 =([\root 3 \of {0.35}, \root 3 \of {0.40}],[\root 3 \of {0.05},\root 3 \of {0.30}])$ be two IVF-FNs. Then

1. By means of Rani and Mishra’s SCF [53] of IVFFNs given in Eq. (2),$\ H(\alpha _3)=H(\alpha _4)=1.100$, but $\alpha _3$ and $\alpha _4$ are two different IVFFNs. It is clear that Rani and Mishra’s SCF [53] cannot distinguish between the two IVFFNs.
2. Based on our proposed new SCF shown in Eq. (21),$\ N(\alpha _3)=0.896$ and $N(\alpha _4)=1.049$. Because$\ N(\alpha _3)=0.896 < N(\alpha _4)=1.049$, the preference ordering between the two IVF-FNs obtained by our proposed new SCF is “$\alpha _3 < \alpha _4$”.

#### Example 7

Let $\alpha _5 =([\root 3 \of {0.25},\root 3 \of {0.40}],[\root 3 \of {0.30},\root 3 \of {0.35}])$ and $\alpha _6 =([\root 3 \of {0.30},\root 3 \of {0.50}],[\root 3 \of {0.05},\root 3 \of {0.15}])$ be two IVF-FNs. It can be determined that

1. Based on Jeevaraj’s SCF [25] of IVFFNs presented in Eq. (3), it obtains $\ P(\alpha _5)=P(\alpha _6)=0.050$, but $\alpha _5$ and $\alpha _6$ are two different IVFFNs. It is clear that Jeevaraj’s SCF [25] has the shortcoming that it cannot identify the preference ordering of the two IVFFNs in this situation.
2. Using our proposed SCF, it follows that $N(\alpha _5)=0.924$ and $N(\alpha _6)=1.105$. Because $N(\alpha _5)=0.924 < N(\alpha _6)=1.105$, the preference ordering between the IVFFNs$\ \alpha _5$ and $\alpha _6$ obtained by our proposed new SCF is “$\alpha _5 < \alpha _6$”.

#### Example 8

Let $\alpha _7 =([\root 3 \of {0.05},\root 3 \of {0.25}],[\root 3 \of {0.45},\root 3 \of {0.60}])$ and $\alpha _8 =([\root 3 \of {0.10},\root 3 \of {0.40}],[\root 3 \of {0.25},\root 3 \of {0.30}])$ be two IVF-FNs. It can be concluded that

1. By Jeevaraj’s SCF [25], it is achieved that $C(\alpha _7)=0.175$, $C(\alpha _8)=0.175$. It is clear that Jeevaraj’s SCF [25] cannot distinguish between any two IVFF-Ns. In other words, Jeevaraj’s SCF cannot identify the preference ordering of IVFFNs $\alpha _7$ and$\ \alpha _8$ in this situation.
2. Based on our SCF, we can get $N(\alpha _7)=0.533$ and$\ N(\alpha _8)=0.788$. Because $N(\alpha _7)=0.533 < N(\alpha _8)=0.788$, the preference ordering between the two IVFFNs is “$\alpha _7 < \alpha _8$”.

#### Example 9

Let $\alpha _9 =([0.20,0.40],[0.20,0.40])$ and $\alpha _{10} =([0.30,0.50],[0.30,0.50])$ be two IVFFNs. It can be obtained that

1. Based on Rani et al.’s SCF [52] which is shown in Eq. (5), then it obtains $R(\alpha _9)=0$, $\ R(\alpha _{10})=0$. Surely, Rani et al.’s SCF [52] cannot distinguish the two IVFFNs.
2. Applying our proposed new SCF, we can get$\ N(\alpha _9)=0.331$ and $N(\alpha _{10})=0.464$. Therefore “$\alpha _9 < \alpha _{10}$”.

#### Example 10

Let $\alpha _{11} =([0.01,0.04],[0.19,0.75])$ and $\alpha _{12} =([0.09,0.09],[0.51,0.84])$ be two IVFFNs. It can be derived that

1. Using Chen and Tsai’s SCF [54] described by Eq. (6), it is easily obtained $\ G(\alpha _{11})=0.850$, $G(\alpha _{12})=0.850$. It is clear that Chen and Tsai’s SCF [54] cannot distinguish the two IVFFNs. Chen and Tsai’s SCF [54] has the weakness that it cannot identify the preference ordering of IVFFNs $\alpha _{11}$ and $\alpha _{12}$ in this situation.
2. Based on our proposed new SCF, $N(\alpha _{11})=0.022$ and$\ N(\alpha _{12})=0.077$. It is clear that “$\alpha _{11} < \alpha _{12}$” by our score function.

**Table 4:** The results of whether distinguishing different IVFFNs by six existing SCFs and our SCF

IVFFNS	Score functions (SCFs)						
	$M(\alpha )$ [53]	$H(\alpha )$ [53]	$P(\alpha )$ [25]	$C(\alpha )$ [25]	$R(\alpha )$ [52]	$G(\alpha )$ [54]	$N(\alpha )$ (Our proposed)
$\alpha _1=([\root 3 \of {0.40},\root 3 \of {0.60}],[\root 3 \of {0.15},\root 3 \of {0.20}])$	N	Y	Y	Y	Y	/	Y
$$\alpha _2=([\root 3 \of {0.35},\root 3 \of {0.45}],[\root 3 \of {0.05},\root 3 \of {0.10}])$$							
$\alpha _3 =([\root 3 \of {0.20},\root 3 \of {0.40}],[\root 3 \of {0.15},\root 3 \of {0.35}])$	Y	N	Y	Y	Y	/	Y
$$\alpha _4 =([\root 3 \of {0.35},\root 3 \of {0.40}],[\root 3 \of {0.05},\root 3 \of {0.30}])$$							
$\alpha _5 =([\root 3 \of {0.25},\root 3 \of {0.40}],[\root 3 \of {0.30},\root 3 \of {0.35}])$	Y	Y	N	Y	Y	/	Y
$$\alpha _6 =([\root 3 \of {0.30},\root 3 \of {0.50}],[\root 3 \of {0.05},\root 3 \of {0.15}])$$							
$\alpha _7 =([\root 3 \of {0.05},\root 3 \of {0.25}],[\root 3 \of {0.45},\root 3 \of {0.60}])$	Y	Y	Y	N	Y	/	Y
$$\alpha _8 =([\root 3 \of {0.10},\root 3 \of {0.40}],[\root 3 \of {0.25},\root 3 \of {0.30}])$$							
$\alpha _9 =([0.20,0.40],[0.20,0.40])$	Y	Y	Y	Y	N	Y	Y
$$\alpha _{10} =([0.30,0.50],[0.30,0.50])$$							
$\alpha _{11} =([0.01,0.04],[0.19,0.75])$	Y	Y	Y	Y	Y	N	Y
$$\alpha _{12} =([0.09,0.09],[0.51,0.84])$$							

In conclusion, Table 4 shows whether distinguishing between different IVFFNs by six existing SCFs and our SCF on six groups, where N denotes that the score function can not distinguish two IVFFNs, Y indicates that the score function can distinguish two IVFFNs and “/” denotes that the score function is not involved in the operation. From Table 4, it is apparent that our suggested new SCF could address the shortcomings of Rani and Mishra’s [53], Jeevaraj’s [25], Rani et al.’s [52], and Chen and Tsai’s [54], which in some situations cannot distinguish between two IVFFNs. That is, our SCF can identify two IVFFNs in any situations.

To further demonstrate the superiority of our proposed score function, the concept of differentiation rate is introduced as a measure of the goodness of the score function. The larger the differentiation rate, the better the score function in distinguishing any two IVFFNs. The differentiation rate (DR) is calculated as follows.

26 $$\begin{aligned} \begin{aligned} DR = \frac{S}{N} \times 100 \%, \end{aligned} \end{aligned}$$

where *N* is the number of IVFFNs groups to be distinguished and *S* represents the number of IVFFNs groups that can be distinguished by the score function.

**Fig. 2:**
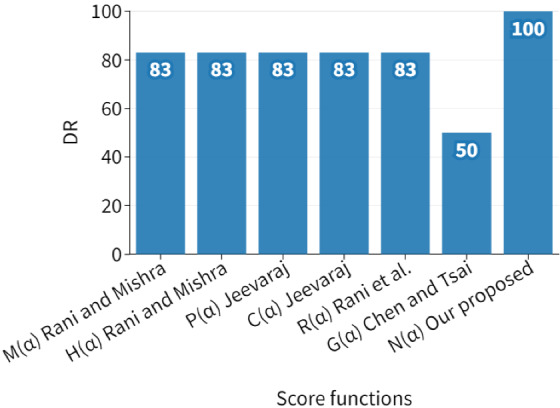
Comparison of the differentiation rates of six existing SCFs and our proposed SCF

From Table 4, it can be concluded that the DR of Rani and Mishra’s SCF $M(\alpha )$ [53] is equal to$\ \frac{5}{6} \times 100 \% =83 \%$, Rani and Mishra’s SCF$\ H(\alpha )$ [53] is equal to $\frac{5}{6} \times 100 \% =83 \%$, Jeevaraj’s SCF $P(\alpha )$ [25] is equal to $\frac{5}{6} \times 100 \% =83 \%$, Jeevaraj’s SCF $C(\alpha )$ [25] is equal to $\frac{5}{6} \times 100 \% =83 \%$, Rani et al.’s SCF$\ R(\alpha )$ [52] is equal to $\frac{5}{6} \times 100 \% =83 \%$, Chen and Tsai’s SCF $G(\alpha )$ [54] is equal to $\frac{1}{2} \times 100 \% =50 \%$ and the DR of our proposed SCF $N(\alpha )$is equal to$\ \frac{6}{6} \times 100 \% =100 \%$.

As can be seen from Fig. 2, our proposed score function has the highest DR, so our score function has the highest differentiation rate in distinguishing different IVFFNs compared with existing score functions. In other words, our proposed score function has the best ability to distinguish arbitrary IVFFNs.

### The proposed MADM method vs. two existing MADM approaches about recognition index

In order to better evaluate the performance of the MADM model, the recognition index (RI) is introduced as the evaluation index of the MADM model. The higher the recognition index of the model, the better the performance of the model. Recognition index is defined as follows:

27 $$\begin{aligned} \begin{aligned} RI = \frac{k}{m} \times 100 \% \end{aligned} \end{aligned}$$

where $i=1,2,...,m$, *m* is the number of alternatives and *k* is the number of alternatives that can be distinguished. Through Case 1, it can be seen that the the approach of Chen and Tasi [54] cannot distinguish the preference orderings of alternatives.

**Fig. 3:**
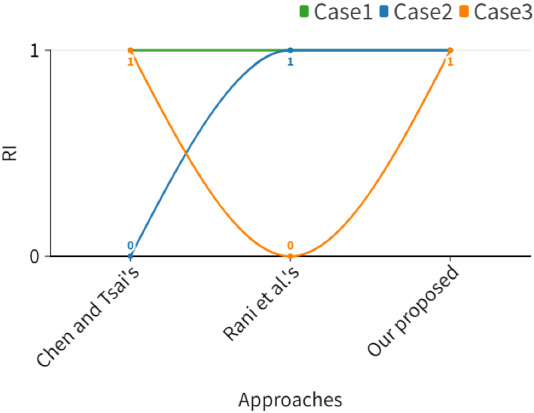
Recognition index comparison with existing approaches

**Table 5:** Division by zero on the two existing methods and our method

	Chen and Tsai’s approach [54]	Rani et al.’s approach [52]	Our proposed approach
Case 1	N	N	N
Case 2	N	N	N
Case 3	N	Y	N
Example 4.1	N	N	N
Example 4.2	N	Y	N

Here, RI is proposed to evaluate the performance of the MADM model. As can be seen from Fig. 3, the RI of the MADM model of Chen and Tsai [54] is equal to 0 in Case 2. Besides, it can be concluded that Rani et al.’s MADM model [52] also cannot distinguish alternatives in Case 3, therefore, the RI of Rani et al.’s MADM model [52] is also equal to 0. Apparently, the greater the RI and the performance of the MADM model is better. And the RI of our proposed MADM is equal to $100 \%$ in Case1, Case2 and Case3.

### Our MADM method vs. the two existing approaches about error rate of division by zero

This part mainly focuses on the comparison of the proposed MADM algorithm with the two existing methods from the perspective of the error rate of division by zero. The lower the error rate of the methods, the better the performance of the models. Here the error rate of division by zero as follows:

28 $$\begin{aligned} \begin{aligned} ER = \frac{t}{y} \times 100 \%, \end{aligned} \end{aligned}$$

where *y* is the number of MADM cases, *t* is the number of cases that have this error of dividing by 0 when using MADM methods for processing MADM applications.

When the MSD of the attribute is equal to NMSD, the method of Rani et al. suffers from the division by zero error, which leads to the inability to obtain the preference orderings of the alternatives. In Table 5, *Y* indicates that this MADM model has the problem of dividing by zero, and *N* indicates that the model does not have the problem of dividing by zero. It is clear that the ER of Rani et al.’s method is equal to $\frac{2}{4} \times 100 \%=50 \%$, the ER of Chen and Tsai’s approach is equal to $\frac{0}{4} \times 100 \%=0$ and the ER of our proposed approach is also equal to$\ \frac{0}{4} \times 100 \%=0$. Therefore, our proposed method and Chen and Tsai’s MADM approach do not have the error of dividing by 0.

## Conclusion

In this paper, a new score function is proposed that can overcome the shortcomings of the existing score functions in Rani and Mishra [53], Jeevaraj [25], Rani et al. [52] and Chen and Tsai [54], which cannot distinguish between two IVFFNs in some situations. Based on our proposed score function, a new MADM approach is constructed that can address the deficiencies that (1) Some existing MADM approaches [52, 54] cannot determine the preference orderings of alternatives in some situations; (2) The MADM approach in Rani et al. [52] has the disadvantage of dividing by zero in the process of decision computation, which prevents the decision-maker from obtaining the preference orderings of the alternatives. Three practical examples illustrate the rationality and superiority of our proposed MADM method. In Case 2, our proposed method has a higher recognition index than Chen and Tsai’s [54] MADM approach. Compared to the approach of Rani et al. [52], our proposed method has a lower error rate in Case 3. Therefore, our proposed MADM approach handles the MADM problem in the interval-valued Fermatean fuzzy environment more effectively.

Nevertheless, the weight of each attribute is given in advance by experts, our approach does not discuss how to determine an appropriate value. In future work, we will discuss how to determine weights, and following the idea of this paper, we can extend our proposed MADM approach to other fuzzy environments, such as hesitant Fermatean fuzzy environment [59], type-2 fuzzy environment [60–62] and type-3 fuzzy environment [63, 64], etc.

## Data Availability

Enquiries about data availability should be directed to the authors.
